# Unique secretory lysosomal tau species are linked to dementia rate in Alzheimer's patients

**DOI:** 10.1002/alz70856_102903

**Published:** 2025-12-26

**Authors:** Preeti Sharma

**Affiliations:** ^1^ Centre for Brain Research, Bangalore, Karnataka, India

## Abstract

**Background:**

The progression of tau pathology in AD follows a predictable pattern known as the Braak or NFT staging system, which correlates with the clinical severity of dementia. The evidence suggests that tau behaves in a prion‐like manner, propagating from one brain region to another and spreading pathology throughout the brain. This spreading of tau aggregates contributes to the worsening cognitive decline observed in AD patients, making tau a critical target for understanding disease progression and identifying potential therapeutic strategies. However, the molecular mechanisms that underlie tau aggregation and propagation remain poorly understood.

**Method:**

Human autopsy brain tissue (frozen) from the prefrontal cortex were used in this study. All the samples are from Braak stage VI with different CDR score. The lysosomal fraction was isolated from human brain tissue from the prefrontal cortex (Brodmann area 10).

**Result:**

We demonstrate that cognitively resilient AD patients, characterized by reduced tau pathology, maintain lysosomal integrity, whereas cognitively vulnerable AD patients, exhibiting greater tau pathology, display compromised lysosomes. Lysosomes in cognitively vulnerable AD brains contain partially digested, seed‐competent pathogenic tau proteins. These proteins primarily consist of the amyloidogenic core, with peripheral regions degraded. The partially digested tau species are secreted, facilitating the spread of tau pathology and contributing to severe cognitive decline. We have demonstrated that tau aggregates can be secreted extracellularly via lysosomal exocytosis, which may facilitate the spread of tau pathology across different brain region

**Conclusion:**

In conclusion, this study underscores the pivotal role of lysosomal integrity in modulating tau pathology and cognitive outcomes in AD. Dysfunctional lysosomes, marked by altered protein expression, pH dysregulation, and tau accumulation, can drive tau propagation and severe cognitive decline. Targeting lysosomal exocytosis pathways offers a promising avenue for mitigating tau pathology and promoting cognitive resilience in Alzheimer's diseases.

